# Variation of *Jatropha curcas* seed oil content and fatty acid composition with fruit maturity stage

**DOI:** 10.1016/j.heliyon.2020.e03285

**Published:** 2020-02-01

**Authors:** Mbako Jonas, Clever Ketlogetswe, Jerekias Gandure

**Affiliations:** Department of Mechanical Engineering, University of Botswana, Gaborone, Botswana

**Keywords:** Agricultural engineering, Chemical engineering, Biochemical engineering, Biofuel, Plant biology, Jatropha, Biodiesel, Fruit maturity stage, Seed oil content, Fatty acid composition

## Abstract

Seed oil production in Jatropha seeds through different maturity stages have been investigated. In order to meet the high demand of oil (feedstock) for large scale biodiesel production, increasing oil content or output in Jatropha seeds is required. Jatropha fruits were harvested at four different maturity stages and the seeds were analysed for oil content. The seed oil was analysed for fatty acid profile. Results from four different geographical locations investigated namely; Mmadinare, Thamaga, Maun and Shashe, have shown a similar trend in lipid accumulation in Jatropha seeds as the fruits mature from green to brown dry. However, maximum oil content in seeds varies with geographical location. Accumulation of oil in Jatropha seeds during maturation follows a parabolic trend and reaches its peak when fruits are yellow. Oil yield in Jatropha seed kernels ranges from 38.7% to 45.8% for the four maturity stages investigated. Overall results have revealed that harvesting Jatropha fruits when they are yellow increases seed oil output by 6–9% when compared to harvesting the fruits when they are brown dry. There is a relationship between the trend in fatty acid composition in Jatropha seed oil and seed oil content trend during fruit maturation. Based on the trend of unsaturated fatty acids in Jatropha seed oil, particularly linoleic and oleic acids, it can be deduced that reduction of seed oil content from yellow brown to brown dry stage is a result of breakdown of some of the unsaturated fatty acids.

## 1 Introduction

Global energy demand is on the rise, and most of this energy (over 80%) comes from fossil fuels [1, 2]. However, fossil fuels are finite and face depletion soon. On the other hand, the combustion of fossil fuels such as petro-diesel and many others such as coal and natural gas contributes significantly to greenhouse gas (GHG) emissions resulting in climate change (global warming). Therefore, alternative energy sources are needed, and renewable energy is the answer. Biodiesel is one of the renewable and clean-burning fuels, which can be used in diesel engines [3, 4, 5]. Among the seed oil producing plants, Jatropha seed oil has emerged as one of the promising feedstock for commercial biodiesel production. This is mainly due to the fact that *Jatropha curcas* oil is non-edible therefore has no competition with food demand as it is the case when food crops such as rapeseed and sunflower which are used as feedstock for production of biodiesel [5, 6]. Biodiesel has received growing interest in the past years in an effort to reduce greenhouse gas emissions. Availability of enough feedstock still remains a challenge in large scale biodiesel production. Increasing oil output from oil-bearing plants such as Jatropha can help meet the demands of large scale biodiesel production.

*Jatropha curcas* plant has a good adaptation to a large variety of soil and climatic conditions [7, 8, 9, 10]. It is a perennial plant that can grow in marginal land and a quick maturing plant species that starts bearing fruits within a year of its planting [11, 12]. It is for these reasons that previous researchers believe that *Jatropha curcas* is one of the best candidates for commercial biodiesel production. Increasing oil output from the seeds is one of the factors that can make commercial biodiesel production from the plant economically viable. Therefore, harvesting Jatropha fruits/seeds when oil content is maximum would increase overall oil output. Higher seed oil yield may increase the economic viability of Jatropha as a feedstock for biodiesel production, therefore harvesting fruits/seeds when oil content is maximum is necessary [13, 14]. Dranski, et al., (2010), investigated the effect of maturation of Jatropha fruits on oil content in seeds. The authors reported variation of oil yield with maturity of fruits and seeds undergo both physical and chemical changes [15]. However findings on yield trends with fruit maturity differ [11, 12, 16, 17].

According to previous investigations, there is no clear trend on accumulation of oil in Jatropha seeds during their various maturation stages, therefore more research is still required to establish the best phase/stage to harvest jatropha seeds for optimal oil production. This study investigates how lipid content in Jatropha seeds varies during various maturation stages of fruits/seeds. The seeds were harvested in four different geographical locations in Botswana. Consequently, maturity stage at which oil content in seeds is maximum has been identified. This study further investigates the influence of fruit maturity stage on fatty acid composition of Jatropha seed oil.

## 2 Materials and methods

**Figure 1:**
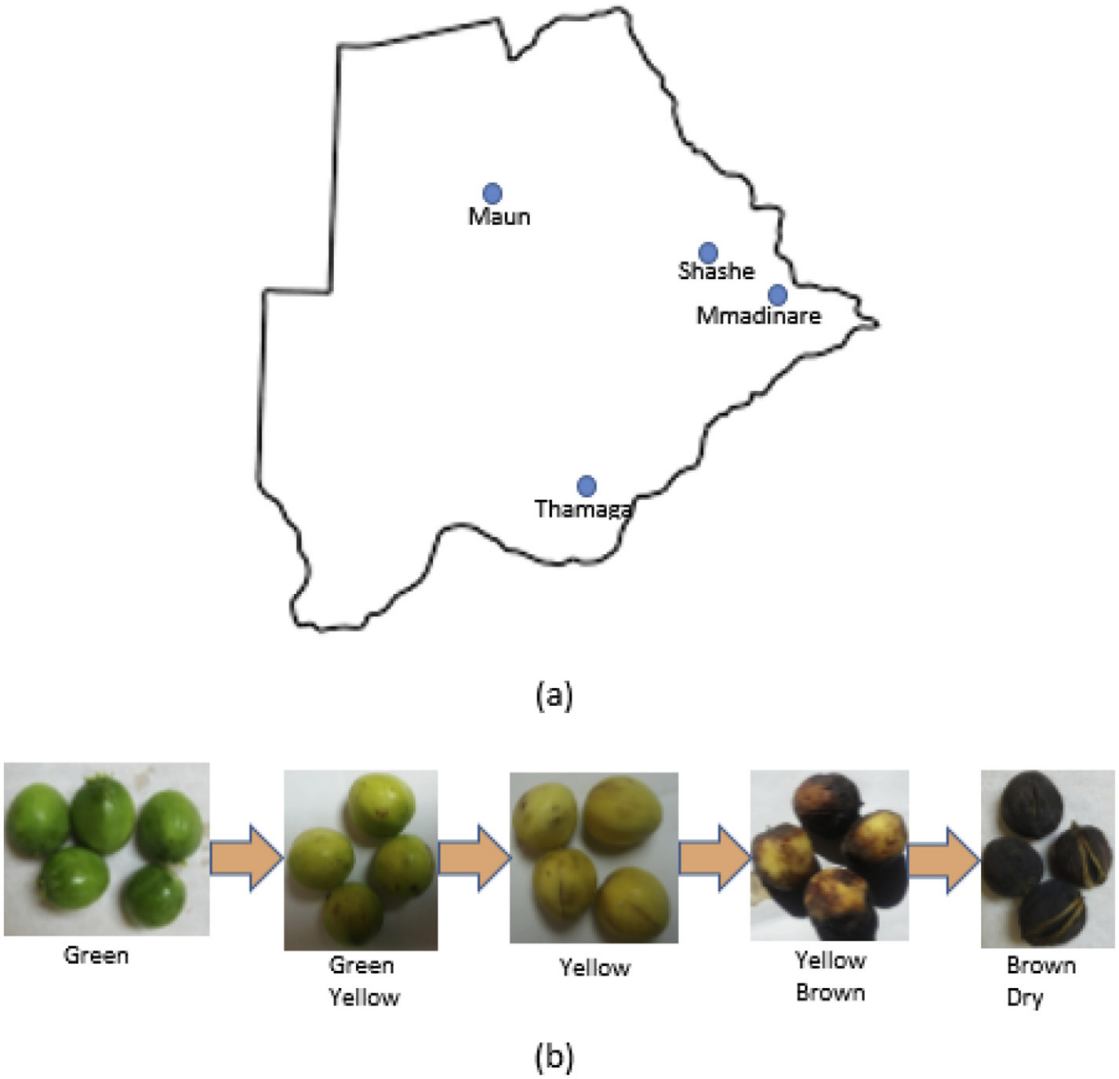
(a) Geographical locations where Jatropha fruits were harvested in Botswana and (b) Jatropha fruits harvested at different maturity stages.

Jatropha fruits and seeds used in this study were harvested at different maturity stages (Green yellow, yellow, yellow brown and brown dry), Figure 1 (b), from four different geographical locations in Botswana, namely; Thamaga (24.72° S latitude, 25.53° E longitude), Maun (19.98° S latitude, 23.42° E longitude), Mmadinare (21.8811^o^S latitude, 27.7514^o^E longitude) and Shashe (21.433^o^S latitude, 27.450^o^E longitude), Figure 1 (a). The fruits were hand-picked from the parent plant. Hand picking allows accurate maturity selection since Jatropha fruits do not mature at the same time. Jatropha fruits show different maturity degrees within the same plant and bunch, therefore using time factor to measure Jatropha fruit maturity is unreliable and inaccurate.

### 2.1 Drying of seeds

Seeds were removed from the fruits within 24 h of harvesting and dried naturally for 10 consecutive days. Seeds were dried in open shade (not direct sunlight to avoid possibility of degradation) in a well-ventilated area at an average temperature of 25 °C. A sample of 100g of seeds from each batch was monitored for change in weight on daily basis. Weighing of seeds was carried out using Shimadzu AW320 analytical balance with a precision of ±0.0001g. The seeds were considered dry when the reduction in weight remained constant. After drying, Jatropha seeds from each maturation stage was analysed for oil content.

### 2.2 Determination of seed oil content

Seed kernels were ground into powder using Mellerware Aromatic Grinder (29105). About 2g of grounded seed kernel was placed in ANK/XT4 filter bag and the filter bag was sealed. The samples in filter bags were dried in an oven at 105 °C for 3 h. The samples were then allowed to cool to room temperature for 15 min in a desiccator. After drying, the samples in filter bags were weighed using Adam Equipment Analytical Balance (AAA 250L). Oil was extracted from the samples using Ankom XT15 extractor. Petroleum ether (chromatography grade) was used as a solvent for the equipment. Sample bags were placed onto the bag holder then put into the Teflon insert. The Teflon insert was put back into the extraction vessel and locked in place into the instrument. The extraction temperature was set at 90 °C and extraction time at 60 min following the manufacturer's recommendation for the use of the instrument. The extraction process was started with the instrument in automatic operation such that after the set time had elapsed, the extraction process stopped automatically. The extraction vessel was removed together with the Teflon insert. The filter bags were then dried in an oven at 105 °C for 30 min to remove residual solvent. The samples were placed in a desiccator for 15 min to cool to room temperature. Each filter bag was re-weighed. The percentage oil content in Jatropha seeds was determined by recording the weight of dried seeds before extraction of the oil then re-weighing the seeds (seed cake) after oil extraction. The procedure was repeated three times for each sample then calculated the average value. Oil yield was then calculated using Eq. (1).

(1) $$\;\%\;Oil\;yield=\frac{W_{2}-\;W_{3}}{W_{1}}\times 100\%$$

Where:

W_1_ = Original weight of sample
W_2_ = Weight of pre-extraction dried sample + filter bag
W_3_ = Weight of dried sample + filter bag after extraction

### 2.3 Determination of fatty acid composition

Fatty acid composition of Jatropha seed oil and derived biodiesel from four different fruit maturity stages was determined using Agilent Technologies GC System 7890A gas chromatograph (GC) according to test method ASTM D6584. The instrument was equipped with an automated injector and HP-5MS capillary column (30 m × 250 μm x 0.25μm). The gas chromatograph was connected to a mass spectrometer (Agilent Technologies 5975C). The fatty acids in the seed oil were converted to methyl esters (biodiesel) by transesterification process [18] before they were injected into the gas chromatograph to improve their volatility. Helium was used as carrier gas. The carrier gas was set at a flow rate of 64 mL/min and pressure of 72kPa and according to manufacturers' specification. The automated injector was set to inject 1μL of sample. The injector and detector were set to operate at a temperatures of 325 °C. The process was set to proceed as follows, initial oven temperature was 100 °C for 4 min. Thereafter, it was increased at a rate of 6 °C per minute to 235 °C, then 10 °C per minute to 300 °C for 8 min. The average run time was about 40 min.

### 2.4 Statistical analysis

The effect of fruit maturity on Jatropha seed oil content was tested using one-way analysis of variance (ANOVA). The statistical analysis was performed using SPSS version 20 software. The means were compared at a significance level of 5% (α = 0.05).

## 3 Results and discussions

### 3.1 Oil accumulation in Jatropha seeds

**Figure 2:**
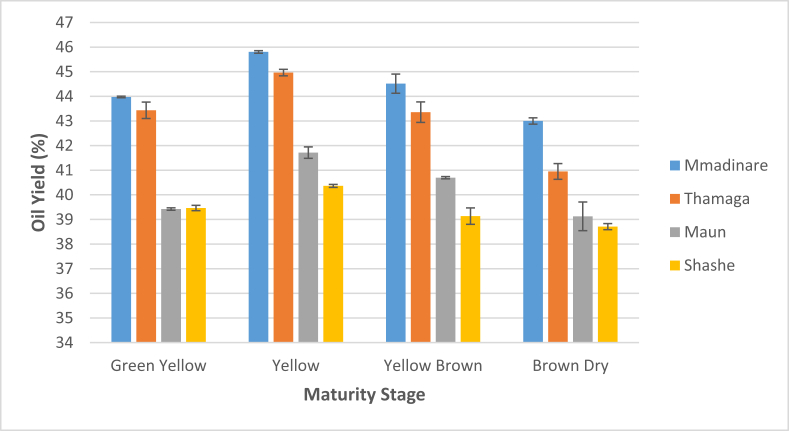
Variation of seed oil yield of Jatropha fruits harvested at four different maturity stages from four different geographical locations.

**Figure 3:**
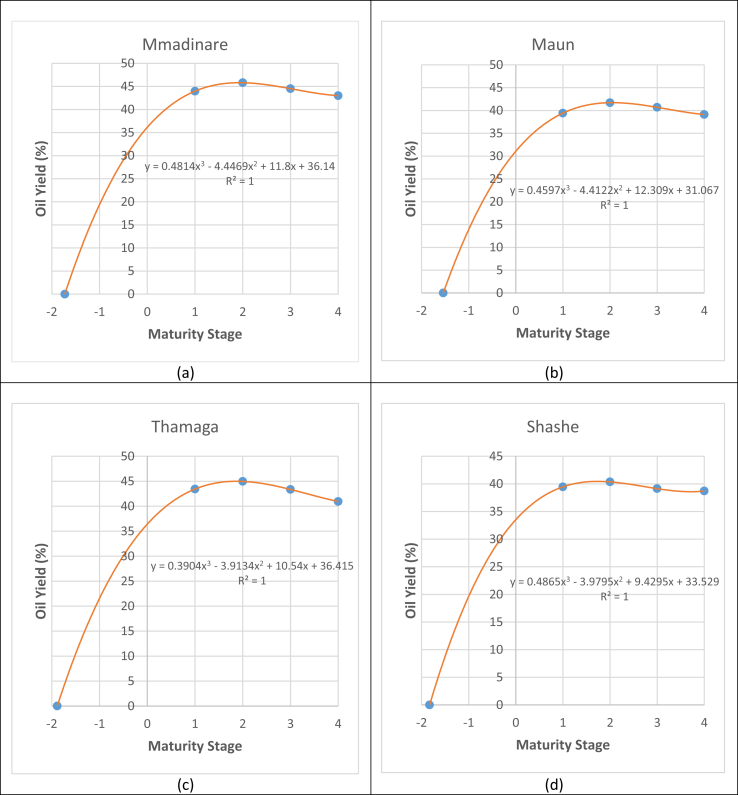
Accumulation of oil in Jatropha seeds from various geographical locations, (a)Mmadinare, (b)Maun, (c)Thamaga and (d)Shashe, from zero up to the final maturity stage (brown dry).

Oil yield in Jatropha seed kernel ranges from 38.7 to 45.8% for the four maturity stages and four different geographical locations investigated as shown in Figure 2. Jatropha seeds harvested in Mmadinare area recorded relatively highest oil yield of 45.8% from yellow fruits. Seeds from Shashe area recorded least oil yield of 40.4% from the same maturity stage, thus 3.3% less. For the four areas under review, variation of seed oil yield with fruit maturity appears to follow the same trend. Oil yield in Jatropha seeds reaches its peak when the fruit turns yellow, and this applies for all the four areas under review as depicted in Figure 3. Overall, the results in Figure 2 suggest that as the fruit turns brown dry oil content in seeds reduces by about 6–9% which is a significant difference. Therefore, harvesting Jatropha fruits when they are yellow increases oil output by 6–9% as compared to harvesting the fruits on their final maturity stage (brown dry).

Generally, accumulation of oil in Jatropha seeds from the four geographical locations in the present investigation follows the trend of a cubic equation of the form y = Ax^3^-Bx^2^+Cx + D, where A, B, C and D are constants. Solving these equations results in x = 1, 2, 3 and 4 which represents green yellow, yellow, yellow brown and brown dry maturity stages, respectively. When oil yield in seeds is zero (y = 0), the cubic equations gives x-values between -1.5 and -2. Therefore, accumulation of oil in Jatropha seeds during fruit maturation can be measured on a scale of -2 to 4 where -2 indicates the beginning of oil synthesis. Oil accumulation trend in Jatropha seeds from the beginning of oil synthesis up to the final maturity stage can therefore be predicted according to the trend lines in Figure 3.

Seed oil content increases as Jatropha fruits matures and reaches its peak when the fruit turns yellow. Thereafter, as the fruit matures further seed oil content start to decline gradually till the final maturation stage of the seed. This decline in seed oil content start as the fruit turns yellow brown and declines further as the fruit turns brown dry. Accumulation of seed oil in Jatropha seeds during maturation follows a parabolic trend as clearly shown in Figure 3. Baud and Lepiniec [19] observed a similar trend when they were studying oil accumulation in maturing seeds of *Arabidopsis thaliana*. They found out that there was a slight fall of the seed oil content in *Arabidopsis thaliana* seeds at the very end of the maturation process. A similar trend was again reported by Eastmond and Rawsthorne [20] who studied oil accumulation in maturing rape seeds. The phenomenon of decline in seed oil content during the final maturation of seeds is still unclear. However, Chia, Pike and Rawsthorne [21] made an effort to investigate this phenomenon after they discovered that there was a loss of at least 10% of storage lipid from *Brassica napus* embryos during the final maturation stage of the seeds. They reported that during the final maturation stage of the seeds, some of the triglycerides undergo β-oxidation (degradation) producing acetyl-CoA (acetyl coenzyme A) hence reduction in overall lipid content. The maturation stage at which lipid content in seeds start to decline is termed ‘Seed desiccation’ [22]. In the present investigation it is appropriate to conclude that Jatropha seed desiccation occurs when the fruit turns yellow brown to brown dry as depicted in Figure 3.

### 3.2 Influence of fruit maturity stage on fatty acid composition

Fractional compositions of fatty acids found in Jatropha seed oil at different fruit maturity stages of Jatropha harvested in Thamaga, Maun, Shashe and Mmadinare areas are shown in Table 1. Linoleic and oleic acids (unsaturated fatty acids) make a significant portion of Jatropha seed oil across all maturity stages of the fruit. For all maturity stages, unsaturated fatty acids make up more than 70% of total fatty acids in Jatropha seed oil. Therefore Jatropha seed oil is more unsaturated. Similar findings were reported by [23, 24, 25] who found out that fractional composition of linoleic and oleic acids in Jatropha seed oil from brown dry fruits contributes over 70% of total fatty acids. Results reveal that these unsaturated fatty acids, particulatly linoleic and oleic acids are the most affected by fruit maturity than the other fatty acids found in Jatropha seed oil. Their fractional composition appear to decrease as fruit maturation advances. The data in Table 1 show that as Jatropha fruits mature from yellow to brown dry (during seed desiccation stage), fractional composition of linoleic acid reduces by 8–9%. Based on this trend of linoleic and oleic acid in Jatropha seed oil, it can be concluded that reduction of seed oil content during seed desiccation stage (from yellow brown to brown dry), as reported earlier in Section 3.2, is a result of breakdown of some of the unsaturated fatty acids. Therefore, there is a relationship between the trend in fatty acid composition in Jatropha seed oil and oil content during the different fruit maturation stages. According to Chia, Pike and Rawsthorne [21] at least 10% of the triglycerides in seed oil undergo β-oxidation (degradation) producing acetyl-CoA (acetyl coenzyme A) during the final maturation stage of seeds hence reduction in overall lipid content in seeds. Unsaturated fatty acids are chemically unstable (easily oxidized), therefore high concentration of such fatty acids in Jatropha seed oil make it susceptible to oxidation and degradation of the oil. Linoleic acid is a polyunsaturated fatty acid (with two double bonds), therefore it is easily oxidized since it is the most unstable fatty acid in Jatropha seed oil. Fractional composition of Palmitic acid, Stearic acid, 11-Hexadecenoic acid and 10-Octadecenoic acid in Jatropha seed oil remain almost constant throughout the maturation stages. Palmitic and Stearic acids are the most stable as compared to the other acids in the seed oil. Their fractional composition remain almost unchanged throughout the maturation of Jatropha seeds.

**Table 1:** Fatty acid profile of Jatropha seed oil at different fruit maturity stages harvested in Thamaga, Maun, Shashe and Mmadinare areas.

Location	Fatty Acid	Maturity Stage/Fractional Composition (%)			
		Green Yellow	Yellow	Yellow Brown	Brown Dry
Thamaga	Linoleic acid	39.766	39.231	38.198	34.789
	Oleic acid	35.323	35.992	37.090	41.079
	Palmitic acid	16.003	16.098	15.617	15.029
	Stearic acid	5.892	5.938	6.284	6.897
	11-Hexadecenoic acid	1.251	1.131	1.164	0.925
	10-Octadecenoic acid	1.765	1.610	1.650	1.282
					
Maun	Linoleic acid	33.111	33.100	33.059	33.072
	Oleic acid	40.196	40.912	41.025	38.945
	Palmitic acid	14.376	14.391	14.895	15.289
	Stearic acid	8.296	8.334	8.348	9.207
	11-Hexadecenoic acid	1.037	0.991	1.211	1.504
	10-Octadecenoic acid	1.591	1.410	1.463	1.982
					
Shashe	Linoleic acid	35.212	34.991	34.211	32.987
	Oleic acid	38.786	38.771	38.111	37.999
	Palmitic acid	13.322	13.445	13.55	13.410
	Stearic acid	9.113	9.100	9.001	9.207
	11-Hexadecenoic acid	1.907	1.002	1.221	1.500
	10-Octadecenoic acid	1.201	0.998	1.473	1.980
					
Mmadinare	Linoleic acid	49.510	55.689	55.063	48.380
	Oleic acid	29.350	21.503	23.191	29.054
	Palmitic acid	16.178	15.851	15.370	15.887
	Stearic acid	4.962	6.176	6.376	6.680
	11-Hexadecenoic acid	-	-	-	-
	10-Octadecenoic acid	-	-	-	-

### 3.3 Statistical analysis

Results of analysis of variance (ANOVA) on effect of fruit maturity on Jatropha seed oil content are presented in Table 2. The effect of fruit maturity on Jatropha seed oil yield is statistically significant because the significance values are less than 0.05 and the calculated F-values are greater than the critical F-value, 4.07. In other words, there is a significant influence of fruit maturity on Jatropha seed oil content. Results of analysis of ANOVA on influence of geographical location on Jatropha seed oil content are presented in Table 3. Influence of geographical location on Jatropha seed oil content is statistically significant. Post Hoc Tukey test results have indicated that variation of seed oil content with geographical location is significant between all the four investigated geographical locations.

**Table 2:** One way analysis of variance on influence of fruit maturity on Jatropha seed oil content.

Parameter	Location	Mean Square	F Test	Significance
Oil Content	Shashe	Between groups: 1.475	13.847	0.002
		Within groups: 0.106		
	Maun	Between groups: 4.280	14.389	0.001
		Within groups: 0.297		
	Thamaga	Between groups: 8.262	4.926	0.032
		Within groups: 1.677		
	Mmadinare	Between groups: 4.148	12.837	0.006
		Within groups: 1.462

**Table 3:** One way analysis of variance on influence of geographical location (Mmadinare, Thamaga, Maun and Shashe) on Jatropha seed oil content.

Parameter	Maturity Stage	Mean Square	F Test	Significance
Oil Content	Green Yellow	Between groups: 17.894	186.682	0.000
		Within groups: 0.096		
	Yellow	Between groups: 20.127	358.010	0.000
		Within groups: 0.056		
	Yellow Brown	Between groups: 18.043	5.940	0.020
		Within groups: 3.038		
	Brown Dry	Between groups: 11.520	32.578	0.000
		Within groups: 0.354		

## 4 Conclusions

Accumulation of seed oil in Jatropha seeds during maturation follows a parabolic trend. Oil yield/content in Jatropha seeds is maximum when fruits are yellow. Harvesting Jatropha fruits when they are yellow increases oil output by 6–9% as compared to harvesting the fruits on their final maturity stage (brown dry). This may significantly increase availability of feedstock for biodiesel production. There is a significant influence of fruit maturity on Jatropha seed oil content. Geographical location also influences seed oil content. Despite having a similar oil accumulation trend for all the investigated geographical locations, Jatropha seed oil content varies from one location to the other. There is a relationship between the trend in fatty acid composition in Jatropha seed oil and oil content trend during the different fruit maturation stages. Fractional composition of unsaturated fatty acids in Jatropha seed oil tend to decrease continuously as fruits mature from green yellow to brown dry resulting in a decline of seed oil content.

## Declarations

### Author contribution statement

Mbako Jonas: Conceived and designed the experiments; Performed the experiments; Analyzed and interpreted the data; Wrote the paper.

Clever Ketlogetswe & Jerekias Gandure: Conceived and designed the experiments; Contributed reagents, materials, analysis tools or data.

### Funding statement

This work was supported by the Ministry of Minerals, Green Technology and Energy Security in Botswana.

### Competing interest statement

The authors declare no conflict of interest.

### Additional information

No additional information is available for this paper.
